# Ultrasound applications in drying of fruits from a sustainable development goals perspective

**DOI:** 10.1016/j.ultsonch.2023.106430

**Published:** 2023-05-04

**Authors:** Fabiano A.N. Fernandes, Sueli Rodrigues

**Affiliations:** a Universidade Federal do Ceará, Departamento de Engenharia Química, Campus do Pici, Bloco 709, 60440-900 Fortaleza, CE, Brazil; b Universidade Federal do Ceará, Departamento de Engenharia de Alimentos, Campus do Pici, Bloco 858, 60440-900 Fortaleza, CE, Brazil

**Keywords:** Ultrasound, Fruit, Drying, SDGs

## Abstract

This review focuses on the many contributions of ultrasound technologies for fruit drying toward the United Nations Sustainable Development Goals (SDG). Along this review, several aspects attained from the application of ultrasound technologies are correlated with the SDGs. The main ultrasonic technologies applied for fruit drying, such as ultrasonic bath, probe ultrasound, air-borne ultrasound air-drying, and ultrasound-assisted contact air-drying, are presented. An in-depth discussion on ultrasound contributions, its advantages, disadvantages, and limitations are made. The effects of ultrasound on water diffusivity in several fruits are presented by correlating this effect with drying time and cost of energy. Ultrasound-assisted fruit drying, like other food processing technologies, directly impacts Zero Hunger, but ultrasound technologies contribute to much more than delivering long shelf-life food. This technology can be used to produce healthy foods and provide well-being, which will be discussed by correlating the effects of ultrasound-assisted air-drying with the concentration of nutritional compounds. Ultrasound-assisted fruit drying reduces wastewater toxicity and energy consumption and improves productivity, potentially improving workplaces and salaries. A walk through the technology is presented from Zero Hunger to No Poverty.

## Highlights

• Ultrasound-assisted fruit drying is connected to 10 Sustainable Development Goals (SDGs).
• Ultrasound (US) processing is directly connected to Zero Hunger and Good Health and Well-Being goals.
• US advantages in reducing processing time fulfill Affordable and Clean Energy goals.
• Advances in industrial technology attained by ultrasound were important to achieve the SDGs.
• Reduction in washing chemical improve Life on Land and Life below Water goals.

## Graphical abstract

**Figure:**
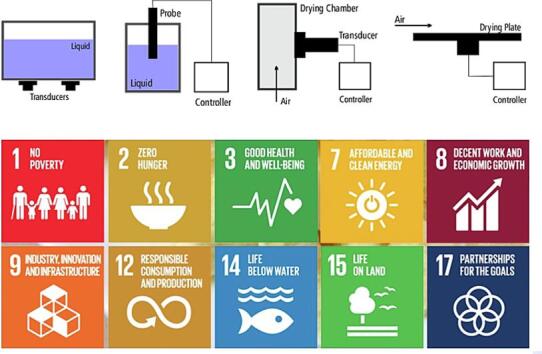

## 1 Introduction

The 2030 Agenda for Sustainable Development, adopted by all member states of the United Nations Organization (UNO), provide a guideline for peace and prosperity for people and the planet. Seventeen Sustainable Development Goals (SDGs) were defined and comprised several urgent calls for action by all countries. These goals consider that poverty can only end if the world can improve health, education, and work conditions, have sustainable economic growth, tackle climate change, and preserve air, land, and oceans.

Although many controversies rely on the transformation of the energy system to stop climate change, many other transformations are required to achieve all SDGs. Substantial changes are needed in the world’s food system. Entities such as the United Nations, the Columbia Center on Sustainable Investment, World Food Programme, World Bank, International Monetary Fund, and others state that the world food system is in crisis. The world is facing severe problems with unhealthy diets, unsustainable food production, food losses, increasing food waste, poverty in farm communities, and an overall vulnerability of the food system to deal with climate changes and other crises [1], [2], [3], [4].

The world food system is highly complex, involving millions of farmers, farm workers, food processing companies, logistic companies, vendors, employees, and consumers. On top of all, there is an extremely varied food production system due to highly diverse food cultures and traditions. All this complex system requires transformation for the world food system to become a sustainable food system. New ways of producing and processing will be necessary, employing more technology. Cleaner, low-energy consuming, and sustainable technologies such as ultrasound, UV light, cold plasma, high-pressure processing (HPP), pulsed electric field (PEF), microwaves, and ozone are suitable for replacing older and less sustainable technologies [5], [6], [7], [8], [9], [10], [11], [12].

Drying has been used since ancestral times to preserve food, fruits included. Dried fruits have been a significant source of nutrients during winter and drought seasons, immensely contributing to combatting hunger. Today, drying is used to preserve fruits during their off-season and for many other purposes, such as easy transportation and incorporation in several food formulations, snacks, and other food products.

Ultrasound applications in the world food system have been studied for at least three decades [5], [13], [14], [15]. Some industries employ ultrasonic processes in cutting, defoaming, extraction, and cleaning [16]. Still, the use of ultrasonics has yet to progress much in industrial drying even though several studies have pointed out its advantages and technical–economic viability. Many industries that produce dried fruits employ low-tech processes that work but are less efficient and consume more energy than needed. Thus, improvement of their technology should be incentivized to meet the SDGs.

Drying is an energy-intensive process having a high cost for the industry. Any technology allowing the reduction of processing time is welcomed by the industry [17]. Ultrasound was first applied to extract pigments and medicinal and bioactive components from plants. Studies carried out with extraction reported that ultrasonic treatment improved the extraction of components from plants due to increased mass transport [14], [18], [19]. The increased diffusion in plants caught the attention of researchers working on drying in the 1990 s, and experiments with ultrasonic pretreatments were carried out to evaluate if this process also increased water mass transfer during the air-drying stage.

Ultrasound was effective in increasing water mass diffusion in fruits, reducing drying time and cost. Thus, much research has been conducted on ultrasound-assisted drying since then. New ultrasonic-based drying equipment and technology were developed, improved, and optimized. And the technology was used to improve the nutritional and sensory aspects of the dried fruit. This review will discuss all these topics, from technology development to sensory improvement, correlating these aspects with sustainable development.

Considering ultrasound applications in fruit drying and the seventeen SDGs, it is easy to correlate it with Goal #2, “Zero Hunger”, because we are dealing with food processing. However, many other goals are fulfilled. This review presents how ultrasound applications in fruit drying are correlated with ten SDGs, presenting the state-of-the-art on this technology and how it contributes towards the SDGs.

## 2 Ultrasound technology applied to fruit drying

Several articles and reviews have focused on the principles and ultrasonic equipment used in food processing [5], [15], [20], [21], [22], [23]. This section will briefly introduce the ultrasound equipment used in fruit drying and the ultrasound-induced mechanisms correlated with fruit processing.

**Fig. 1:**
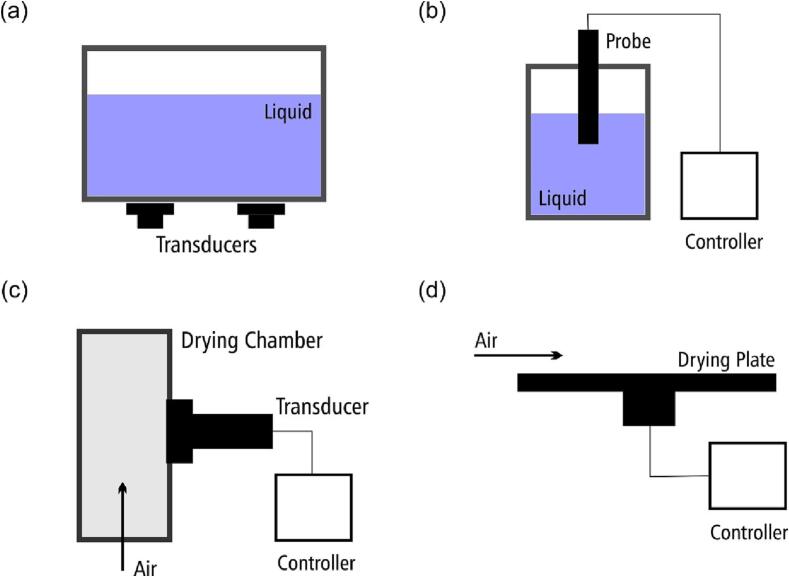
Most common ultrasound systems for fruit drying: (a) bath ultrasound; (b) probe ultrasound; (c) air-borne ultrasonic drier; (d) ultrasonic contact air-drier.

The most common equipment used in fruit processing and fruit drying is bath, probe, air-borne, and direct-contact ultrasounds (Fig. 1). The operation of bath and probe ultrasounds require the application of ultrasound in water, osmotic solution, or other solvents; thus, these equipment are used in ultrasonic pre-treatments. In this case, the fruit is subjected to ultrasound, immersed in a liquid, and afterward dried using conventional drying processes, such as air-, oven-, microwave-, vacuum-, or infrared drying [20], [24], [25], [26]. Air-borne and direct-contact ultrasounds are operated in the air; thus, ultrasound is directly applied during the entire drying operation [27], [28], [29]. Each technology has some advantages and disadvantages, which will be discussed and detailed in Section 8.

The bath ultrasound is the simplest ultrasonic system used in drying processes. The equipment consists of a water bath where the fruit is treated. One or more transducers are placed at the bottom of the bath. Ultrasound is generated by the transducers and transmitted through the water or other liquid to the sample. Bath ultrasound operates in a single frequency with transducers generating ultrasound at frequencies from 21 to 100 kHz, with most common transducers operating at 25, 33, and 45 kHz [16], [30], [31]. Bath ultrasounds operating at different frequencies exist but are harder to find. Special multifrequency bath ultrasounds are found, but they have two or three transducers operating at different frequencies that can be turned on and off, operating at a single or in more than one frequency at the same time. Bath ultrasound outputs low ultrasonic power density (<200 W/L). The main variables that can be changed are the processing time, sample loading (kg/L), and the liquid level, which gives a very slight control of the ultrasonic density [30], [31].

Probe ultrasound is one of the more powerful ultrasounds, enabling ultrasonic power densities above 1000 W/L [30], [31]. Probe ultrasound consists of a controller, a transducer, and a probe. The probe is immersed in a liquid where ultrasound will be transmitted to the fruit being treated. This equipment operated at 18 to 24 kHz in a single frequency. Depending on the manufacturer, probes with several diameters can be used, allowing control of the power density and amplitude of the waves. The power density also can be controlled by setting lower energy output in the controller and by choosing different volumes for the vessel where sonication will be carried out.

Air-borne ultrasound consists of an ultrasound generator, an amplifier, a ring probe, and a drying chamber. In this equipment, ultrasound is amplified and transmitted to a ring probe surrounding the metallic drying chamber, which vibrates, transmitting ultrasound to the fruit placed inside the chamber [22], [32], [33]. Air passes through the drying chamber as in a conventional drier. Most systems operate at a single frequency, but some special multifrequency ultrasound generators can be used, allowing for a change in the operating frequency. This feature comes with a much higher price than single-frequency units. This system permits changing the ultrasonic power, loading, air temperature, and air velocity [34], [35], [36], [37].

Ultrasonic contact air dryers consist of an ultrasound generator, an amplifier, and a probe shaped like a plate. The fruit is placed directly on the vibrating plate, and air passes above the plate. Like air-borne ultrasound, this system allows changing the ultrasonic power, loading, air temperature, and air velocity [38], [39].

Ultrasound technology induces several physical and chemical changes in fruit. The physical changes are mainly caused by cavitation, micro-jet streaming, and the sponge effect [5], [15], [31]. While the chemical changes are mainly caused by the generation of hydroxyl radicals and oxygen peroxide during sonication. Cavitation is the main physical effect observed during ultrasound application, especially in liquids. As an oscillating sound wave passes through a liquid or sample containing liquids, pressure will oscillate, generating bubbles [40]. These bubbles grow due to rectified diffusion until they reach their resonance radius, and then they collapse. The collapse of these bubbles causes localized high peak pressures and temperatures [31], [41]. Cavitation causes the breakdown of organic tissues and therefore is especially useful to sanitize fruits before drying, to inactivate enzymes, to form microscopic channels during fruit pre-treatment, and to reduce mass-transfer resistance between the fruit surface and its environment. Micro-jet streaming is a consequence of cavitation occurring near liquid–liquid or liquid–solid interfaces [42]. When cavitation occurs near an interface, part of the liquid near the bubbles is ejected at high speed forming micro-jet streams that invade the other phase [43]. Such phenomena contribute to the reduction of mass-transfer resistance between the fruit surface and the liquid in which the fruit is immersed. The sponge effect occurs when ultrasonic waves travel through the fruit, causing a rapid alternating compression and expansion of the fruit tissue. This phenomenon was called the “sponge effect” because it can be compared to what happens when a sponge is squeezed and released multiple times [31]. The “sponge effect” contributes toward the formation of microscopic channels inside the fruit and enhances the mass transfer of water from the fruit to the environment. These effects will be correlated with several aspects of fruit drying in the following sections.

The collapse of bubbles during cavitation releases a high amount of energy into the surroundings. Part of this energy is transformed into internal energy in water molecules, which may heat or go through chemical reactions [44], [45]. When the amount of energy is enough to surpass the activation energy for chemical reactions, water is transformed into hydroxyl radicals. These radicals may combine, forming hydrogen peroxide, which is one of the main compounds formed during ultrasound application [44], [45]. Such a reaction can occur in the liquid phase, where the fruit is immersed during ultrasonic pre-treatment and inside the fruit tissue. Hydrogen peroxide and hydroxyl radicals can directly react with compounds of the fruit and activate a series of metabolic or enzymatic reactions in the fruit [46]. The chemical changes induced by ultrasound will be discussed in more detail in Section 6.

**Fig. 2:**
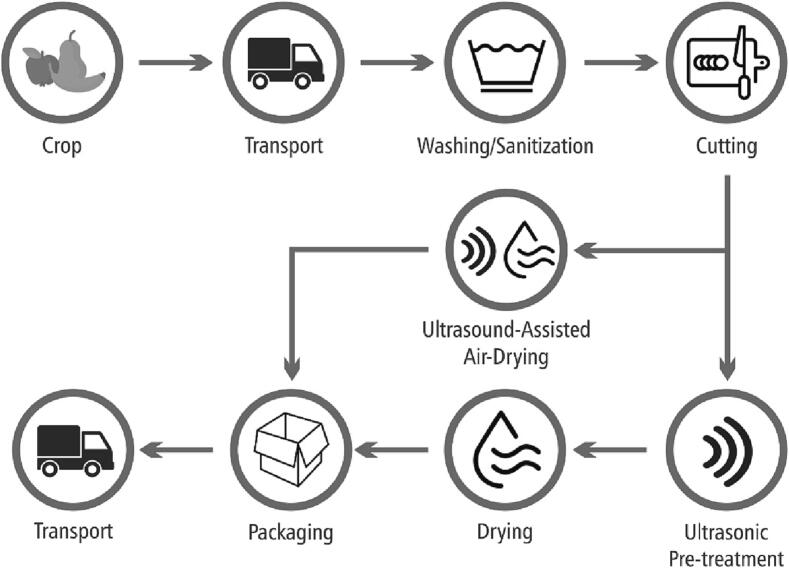
Traditional process stages in a fruit drying facility applying ultrasound-assisted drying technologies.

In a fruit drying facility, the fruit is received, washed, sanitized, cut into smaller pieces if needed, dried, and then packaged (Fig. 2). The drying process can occur in two steps, ultrasonic pre-treatment and drying, or in a single step when applying direct ultrasound-assisted air or contact drying. Ultrasound can be applied during the drying stage and also during the washing and sanitization stages. In the following Sections, we will follow the production pathway linking them with the SDGs.

## 3 Goals #14 and #15 – “Life below Water” and “Life on Land”

As fruit enters a processing facility, it is washed and sanitized to remove dirt and microorganisms. Washing and sanitization are usually done together employing chlorine or other sanitizers. Among all sanitizers, chlorine is the most used since it has good effectiveness against bacteria, mold, yeasts, and viruses and has a meager cost [47]. However, chlorine and other sanitizers are associated with adverse health outcomes. The washing water containing chlorine and other substances is rarely treated and usually ends up in rivers, lakes, and the ocean contributing to pollution and affecting life below water and on land.

Although some European countries have banned the use of chlorine, they still allow the use of other environmentally unfriendly sanitizers. Reducing the amount of chlorine and other sanitizers is an essential contribution to the SDGs, which can be achieved by ultrasound application. Ultrasonic baths have been used in many industries to remove dirt, grease, salts, and other substances from many surfaces [48], [49], [50], [51].

Along with dirt, fruits come to the industry with many endemic pathogens on their surface. Microorganisms such as aerobic bacteria, molds, yeasts, lactic acid bacteria, coliforms, *Pseudomonas*, *Salmonella*, *Escherichia*, and *Listeria* have been reported on the surface of fruits [52], [53], [54], [55], [56]. Much research has been done on the washing and inactivation of microorganisms in fruits by applying ultrasound [52], [53], [54], [55], [56]. Ultrasound inactivates microorganisms by the breakdown of the cell membranes. Cavitation near the surface of the microorganisms tends to rupture the cell membranes resulting in leakage of intracellular material and death of the microorganism. The sponge effect helps to rupture the cell membrane due to the repeating expansion and contraction movement. The production of hydrogen peroxide during sonication theoretically helps in sanitization, but the concentration attained in the process is low, considering the concentration needed for full sanitization.

Unfortunately, ultrasound alone cannot eliminate microorganisms from fruits, except for very few cases. Complete removal or inactivation of microorganisms during the washing and initial sanitization stage is usually achieved with the combination of ultrasound and other methods, such as heat, pressure, and the addition of sanitizers. The efficacy of the process depends on several factors, such as the resistance of the microorganisms to ultrasound, processing time, surface topology, and ultrasonic power density [57], [58], [59].

Washing and sanitization are usually carried out in bath ultrasounds due to their higher loading and processing capacity. Despite their high loading capacity, bath ultrasounds work at ultrasonic power densities between 30 and 200 W/L, which can be considered low compared to what can be achieved with probe ultrasounds (greater than1000 W/L). As such, this process relies more on the sponge effect than on the cavitation effect. Under this condition, ultrasound alone will only be effective on microorganisms very susceptible to ultrasound, such as *Escherichia coli*, *Salmonella enteritidis*, and *Listeria innocua* [54], [56], [60]. These three species were the only ones with a reduction between 4 and 6 log CFU that has been reported when treated solely with ultrasound [54], [56], [60]. *Salmonella enteritidis* counts were reduced by more than 5 log CFU in inoculated Romaine lettuce applying very low ultrasonic power density (30 W/L, 30 min) [60]. *Escherichia coli* and *Listeria innocua* counts were reduced by more than 4 log CFU in inoculated cabbage and lettuce by applying mid-range ultrasonic power density (100 to 300 W/L) [56].

Surface topology and porosity affect the sanitization efficacy of ultrasound, but this factor still needs to be fully understood. Although *Escherichia coli*, *Salmonella enteritidis*, and *Listeria innocua* are very susceptible to ultrasound, the best inactivation results were attained for leaf vegetables (cabbage and lettuce). The surface topology and porosity of fruits tend to reduce the effect of ultrasound in sanitizing these microorganisms resulting in a lower count reduction. While a decrease in *Listeria innocua* of over 4 logs CFU was observed on cabbage, a reduction slightly below 3 logs CFU was observed on blueberries [55].

Processing times between 10 and 60 min have been studied for washing and sanitization in ultrasonic baths. Reductions above 5 logs CFU can be achieved after 15 min of sonication at mid-range ultrasound power density (100 to 300 W/L). Still, more time is required for this level of inactivation depending on the fruit being processed and its microorganism load. The ultrasonic frequency does not seem to have a high effect on sanitization, and baths with frequencies between 25 and 42 kHz could be employed.

Aerobic bacteria, lactic acid bacteria, molds, yeasts, *Enterobacteriaceae*, *Staphylococcus,* and *Pseudomonas,* are not very susceptible to ultrasound, and count reductions below 2 log CFU are generally reported for fruits [52], [53], [61], [62]. Thus, ultrasound alone cannot eliminate microorganisms from fruits and should be combined with other methods.

Ultrasound, however, enhances the effect of several sanitizers, acids, ozone, and antimicrobials reducing the concentration needed in sanitization. Its enhancing power has been verified with chlorine dioxide, hydrogen peroxide, peracetic acid, malic acid, lactic acid, citric acid, acetic acid, ozone, carvacrol, vanillin, citral, sodium hypochlorite, and sodium dichloroisocyanurate [55], [61], [63], [64], [65], [66], [67].

**Fig. 3:**
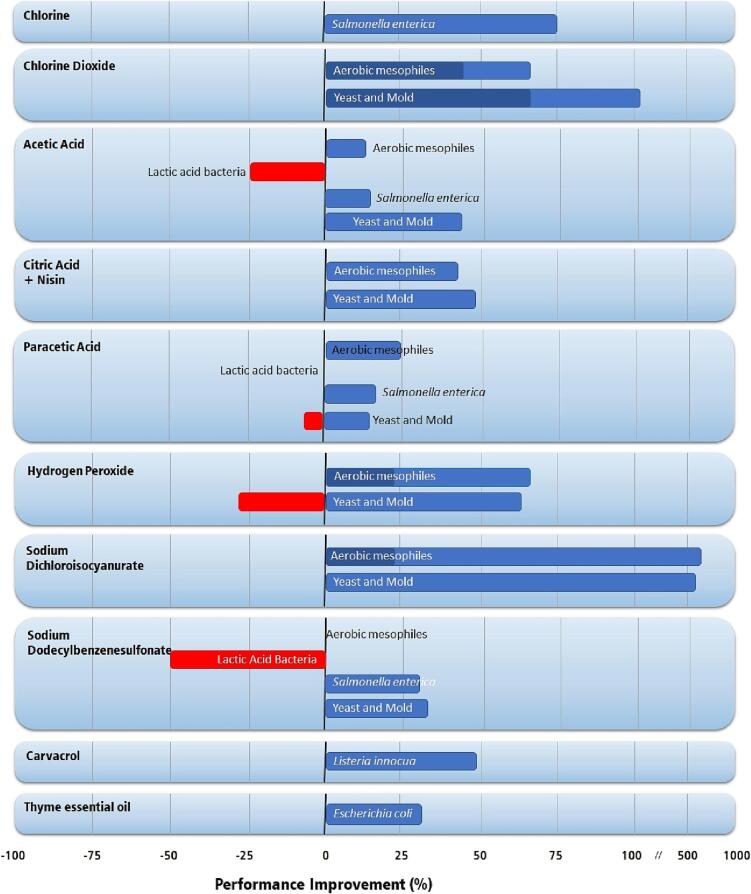
Improvement in the performance of sanitizers by ultrasound application during the initial washing and sanitizing stage of fruits and vegetables. Data were obtained in the literature [61], [63], [64], [65], [68], [69], [71], [55]. For case studies reported by more than one article, dark blue and red bars indicate the minimal values reported for performance improvement.

Fig. 3 presents the improvement in the performance of sanitizers attained in association with ultrasound application. Overall, ultrasound improves the efficacy of sanitizers, with a few exceptions. The gain in the effectiveness of chlorine and chlorine dioxide was high, with most works reporting improvements between 45 and 110% [68], [69]. Hydrogen peroxide has its efficacy enhanced by more than 60% in most reported cases [62], [64], [69], [70], except for the washing and sanitizing of watercress, which presented a lower efficacy against yeast and mold [69]. However, this behavior is strange because ultrasound induces the formation of hydrogen peroxide. The most significant performance improvement occurred with sodium dichloro isocyanurate (up to 660%) [69], [70]. Improvements in acids performances were lower than in chlorines and remained within 15 and 90%, except for lactic acid bacteria, which did not improve its performance [65], [69], [70]. Essential oils, such as carvacrol and thyme essential oil, had their efficacy improved against *Listeria* and *Escherichia coli* [71], [55]*.*

The association between sanitizers and ultrasound was generally more effective for the inactivation of aerobic mesophiles, yeast, molds, *Salmonella*, *Listeria*, and *Escherichia coli*. However, the association was not successful in lactic acid bacteria inactivation. Thus, the microbiota of the fruit being washed and sanitized must be known to ensure good performance in this processing stage.

**Fig. 4:**
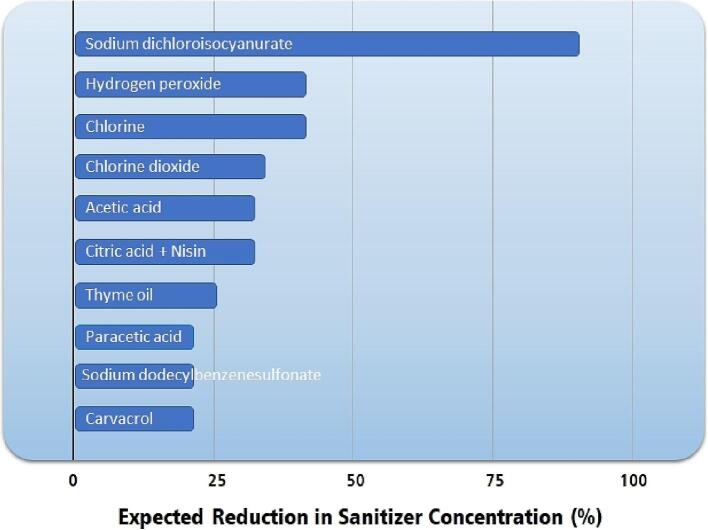
Estimated reduction in sanitizer concentration attained by ultrasound application during the initial washing and sanitizing stage of fruits and vegetables. Data were obtained in the literature [61], [63], [64], [65], [68], [69], [71], [55].

Reduced sanitizer consumption by the fruit processing industry can be directly linked with reducing pollutant emissions. Most sanitizers end up in lakes, rivers, and oceans, affecting the entire aquatic ecosystem. Fig. 4 presents an estimated reduction in sanitizer concentration that can be attained by associating sanitizer and ultrasound application in the initial washing and sanitizing stage.

Applying ultrasound in the washing and sanitization process can reduce more than 75% of the quantity of sodium dichloroisocyanurate and more than 25% of the amount of hydrogen peroxide, chlorine, chlorine dioxide, acetic acid, and citric acid used in washing and sanitizing stages. A reduction of 40% in the concentration of chlorine used in the industry may not be economically attractive because of the low cost of chlorine and the higher cost of energy in some regions of the planet. Still, the association between ultrasound and chlorine implies a significant reduction in the environmental impact of the process.

Thus, ultrasound application in the fruit drying industry helps achieve SDG #14 and #15 by reducing the amount of sanitizers used and discarded in nature.

## 4 Goal #2 – “Zero Hunger”

The SDG #2 sets the need to end hunger, achieve food security and improved nutrition, and promote sustainable agriculture. In 2022 the United Nations stated that wars, conflicts, climate change, endemics, pandemics, and growing inequalities significantly affect food security worldwide. Nowadays, 10% of the world’s population suffers from hunger, and nearly 30% lacks regular access to healthy or nutritious food [72].

The Covid-19 pandemic has compromised food security, and 47% of countries were affected by soaring food prices in 2020 [72], [73]. War and regional conflicts have affected important agriculture-based countries triggering food shortages for the world’s poorest people [74], [75], [76].

Too much food is being lost or wasted in every country every day. According to the United Nations, 13% of the world’s food is lost after harvesting and before reaching retail markets, and these losses occur during harvest, transport, storage, and processing. Another 17% of food is wasted at the consumer level, including our houses, grocery stores, and restaurants. Thus, 30% of all food produced is never eaten [72].

Hunger and undernourishment eradication is a complex problem that cannot be easily solved. It involves food production, logistics, government policies, money, and several other factors [77]. As stated before, 30% of all food produced is never eaten, and about half of that is lost even before it reaches any consumer. Applying simple mathematics, the food produced in the world is enough to end hunger and undernourishment. Unfortunately, more than simple mathematics is needed to solve this problem.

**Fig. 5:**
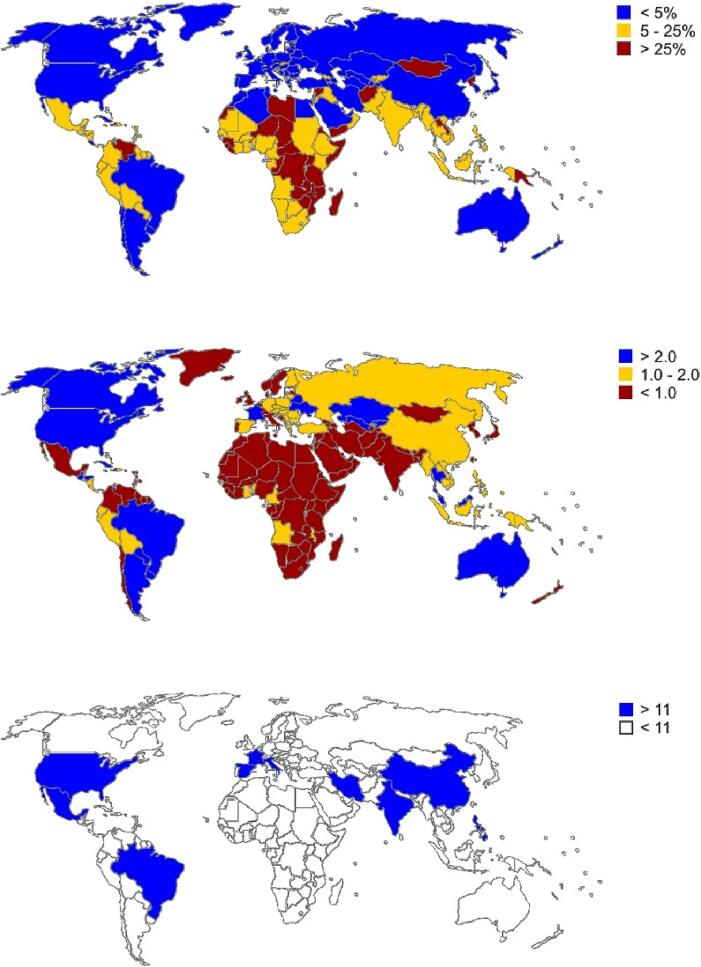
(a) Prevalence of undernourishment in the total population – adapted from the World Food Program hunger map of 2020; (b) Crop production per capita – data of tons of food produced per capita adapted from the Food and Agriculture Organization; (c) Fruit production – data of million tons of fruit production adapted from the Food and Agriculture Organization.

There is a tremendous logistic problem. Comparing the hunger map (Fig. 5a) and the crop production per capita map (Fig. 5b), it is possible to observe that hunger and undernourishment are severe in countries with low crop production per capita. Even in countries with high crop production per capita, hunger and undernourishment are higher in places where food production is scarce, such as in Brazil, where hunger and undernourishment are higher in the Northeast region, where soil and climate conditions do not favor low-tech agriculture. And there are some exceptions, such as Sweden and Japan, which has low crop production per capita but almost no hunger and undernourishment. High-income countries overcome the logistic problem proving that this problem can be minimized. They sell services and industrialized goods and buy most of their food from the producing countries. Low-income countries cannot afford to overcome the logistics problem, and in some countries, the government does not have the will to overcome this problem for several reasons beyond this review's scope. However, the logistic issue must be addressed to end hunger and undernourishment.

Drying is one of the solutions for the logistic problem. Analyzing the food products available in high-income countries with low crop production per capita, it is possible to notice many industrialized products rather than fresh produce. Besides convenience, many industrialized products have a lower cost and a higher shelf life than fresh products, such as fruits and vegetables. The price of fresh products produced abroad increases dramatically due to the logistics cost and losses. For example, in Brazil, papayas cost US$ 1.50/kg and US$ 3.50/unit in Japan (prices on 10/2022). Bananas cost US$ 0.40/kg in Costa Rica and US$ 2.80/kg in Denmark (prices on 10/2022).

Few countries produce enough fruit to supply the world́s needs (Fig. 3c). This means that logistic costs to export fresh fruit to the rest of the world elevate the fruit’s price and generate considerable production losses. Furthermore, as the price is high, consumers want only perfect fruits. Fruits with little imperfections are not exported and are often discarded, not even sold in the local or internal market.

Drying fruits poses a solution to fruits that will not or cannot be sold as fresh produce. Drying increases the shelf life of fruits that can be stored much longer than fresh fruits [78], [79], [80]. Furthermore, it can be consumed in places where fresh produce cannot reach in a reasonable time and would be lost due to deterioration.

Ultrasound technology has been well-studied for fruit drying. Ultrasound can be used as a pre-treatment or during air-drying [22], [27]. The pre-treatment consists of immersing whole or cut fruits in water or an osmotic solution, subjecting ultrasound during the pre-treatment in an ultrasonic bath or probe ultrasound, and then drying the treated fruit [81]. The second option is applying ultrasound during air drying using a special vibrating drying chamber [22].

Independent of the method used, ultrasound-assisted drying relies mainly upon the sponge effect to remove water from the fruit. The forces involved by the sponge effect can be higher than the surface tension that maintains the moisture inside the fruit capillaries, inducing dewatering. The sponge effect also creates microscopic channels inside the fruit tissue structure that ease moisture removal. Water molecules can use these microscopic channels as a preferential pathway to diffuse toward the surface of the fruit, increasing its effective water diffusivity. The ultrasonic waves also reduce the diffusion boundary layer and increase the convective mass transfer in the fruit. Furthermore, water cavitation produced by ultrasound helps remove moisture strongly attached [82].

**Fig. 6:**
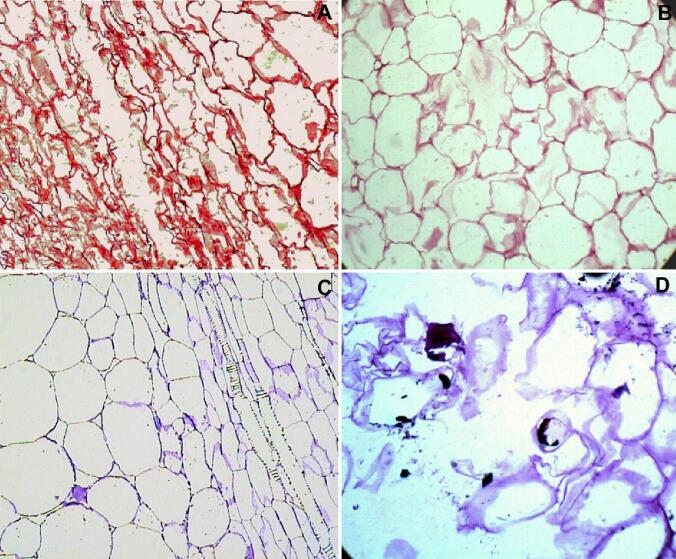
Micrographs of fruit tissue structure presenting microscopic channels created after ultrasonic pretreatment. A. pineapple (*Ananas comosus*); B. papaya (*Carica papaya*); C. melon (*Cucumis melo*); D. sapota (*Manilkara zapota*).

The ultrasonic pretreatment creates microscopic channels inside the fruit tissue, improving water removal (Fig. 6). The format and extension of the microscopic channels depend greatly on the fruit and its original tissue structure. Pineapples (*Ananas comosus*) have thin-walled cells and are very susceptible to the formation of microscopic channels, which are very long. Its formation occurs mainly due to the breakdown of cell walls [83]. Papayas (*Carica papaya*) have thicker cell walls than pineapples which difficult the formation of microscopic channels, which tend to be smaller and more dispersed throughout the tissue structure. Its formation occurs mainly due to the solubilization of the secondary lamella and detachment of neighboring cell walls [84]. Melons (*Cucumis melo*) present an elongation of cells which becomes microscopic channels [85]. The microscopic channels in sapotas occur by the breakdown of its phenolic-dense cells producing large and long channels [86]. The increased water diffusivity in most fruits subjected to ultrasound pretreatment indicates that some sort of microscopic channels is formed in them.

**Fig. 7:**
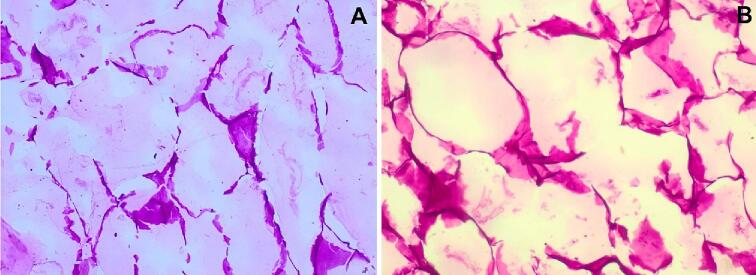
Micrographs of apple (*Malus domestica*) tissue structure presenting microscopic channels created by ultrasonic pretreatment (A) and ultrasonic-assisted air-drying (B).

Microscopic channels are also formed during ultrasound-assisted air-drying. There is scarce information on tissue structure modifications of ultrasonic-assisted air-dried fruits, but a study proved that microscopic channels are also created during this drying technique [27]. The microscopic channels formed during ultrasound-assisted air-drying were smaller and less dispersed in the tissue structure than the microscopic channels formed during ultrasound pre-treatment (Fig. 7). The smaller and less dispersed microscopic channels formed during ultrasound-assisted air-drying are due to the less efficient transmission of ultrasonic waves in the air than in liquids, which attenuates the sponge effect [35].

**Fig. 8:**
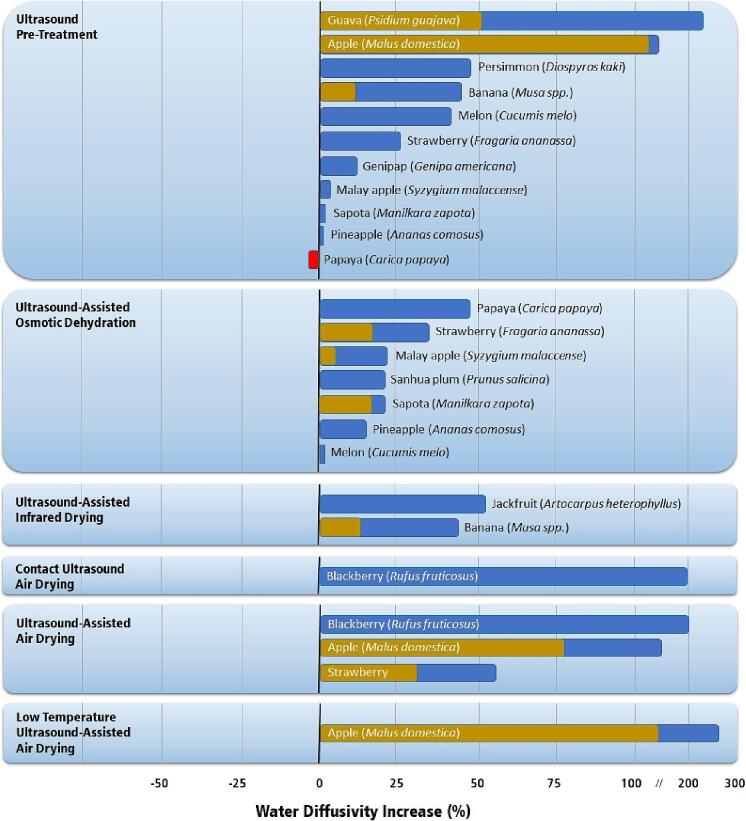
Increase in effective water diffusivity for several fruits and ultrasonic drying processes compared to traditional air drying [24], [27], [34], [37], [39], [81], [83], [84], [85], [86], [87], [88], [89], [90], [91], [92], [93], [94], [95]. Single-colored bars indicate unique values in the literature. Double-colored bars indicate the lowest (dark blue) and highest (light blue) values reported in the literature.

The sponge effect, microscopic channels, and the reduction in the diffusion boundary layer contribute toward a higher water diffusion in fruit subjected to ultrasound than in non-sonicated fruits. Fig. 8 presents the percentual increase in water diffusion for fruits treated by ultrasonic technologies compared to the values attained by conventional air-drying.

Ultrasound pre-treatment consists in immersing the whole or cut fruit in water, subjecting it to ultrasound, followed by conventional air-drying. The increase in water diffusivity observed during the air-drying stage varies considerably depending on the fruit being processed. Guava (*Psidium guajava*) [87] and apples (*Malus domestica*) [27] presented the highest increase in water diffusivity due to ultrasound pre-treatment. Persimmon (*Diospyros kaki*) [88], bananas (*Musa* spp.) [24], [81], melon (*Cucumis melo*) [85], and strawberries (*Fragaria ananassa*) [89] showed an increase higher than 25% in water diffusivity, a value that can be considered satisfactory. Other fruits, such as genipap (*Genipa americana*) [90], Malay apple (*Syzygium malaccense*) [91], sapota (*Manilkara zapota*) [86], pineapple (*Ananas comosus*) [83], and papaya (*Carica papaya*) [84] did not increase considerably the effective water diffusivity; thus, the ultrasound pre-treatment is not recommended for these fruits.

Ultrasound-assisted osmotic dehydration pre-treatment (UAOD) consists in immersing the whole or cut fruit in an osmotic solution, subjecting it to ultrasound, followed by conventional air-drying. The increase in water diffusivity attained by this method was generally smaller than that achieved by ultrasonic pretreatment. The lower increase is mainly due to the saturation of the surface of the fruit with the osmotic solute, usually sucrose or other sugars, such as glucose, fructose, mannitol, and natural syrups [96]. These sugars form a barrier on the surface of the fruit that decreases the apparent water diffusivity. Reasonable water removal is observed (between 10 and 30%) during UAOD, but the effective water diffusivity during air-drying is lower than that attained by ultrasonic pretreatment. However, the water diffusivity after UAOD is higher than the water diffusivity of the untreated fruit.

Although the average water diffusivities observed after UAOD are lower than those after ultrasonic pretreatment, some fruits respond better to UAOD than to ultrasonic pretreatment due to the induced osmotic pressure. For example, papaya (*Carica papaya*) [84], strawberries (*Fragaria ananassa*) [89], Malay apples (*Syzygium malaccense*) [91], and sapota (*Manilkara zapota*) [86] presented higher water diffusivities after UAOD than after ultrasonic pretreatment.

Ultrasound-assisted air-drying (UAAD) consists of applying ultrasound during air-drying using a special vibrating drying chamber. Two different techniques can be applied. The first one consists of a vibrating drying chamber that sonicates the air around the fruit [28], [34], [92], [93], [94]. The second technique consists of a vibrating platform that sonicates the fruit placed over a direct contact platform [39]. The increase in water diffusivity applying the UAAD techniques is usually high, typically exceeding 50%.

Although it seems that UAAD techniques are better than the ultrasonic pretreatments followed by conventional air drying, the advantages of each method should be carefully assessed. The increase in water diffusivity attained by UAAD is usually higher than the achieved by pretreatment. Still, UAAD techniques operate at lower airflow rates because high flow rates disrupt the ultrasonic waves. Thus, the lower water diffusivity of the ultrasonic pretreatments is compensated by higher flow rates. A comparative study with apples showed that processing time was similar when applying either technique [27].

There are many studies on ultrasound-assisted drying in the literature; however, most studies report empirical equations to correlate drying, such as Page, logarithm, Weibull, and other equations. These correlations are valid only for the applied conditions and cannot be used to compare fruits and processes. Studies on drying are highly recommended to report effective water diffusivity rather than simple mathematical correlations.

The importance of increasing the water diffusivity using ultrasound for the SDGs consists in reducing air-drying time. Shorter processes increase productivity and reduce overall costs leading to cheaper products. Hunger affects the poor, which suffer from buying food. Developing processes that can reduce processing costs certainly contribute towards the end of hunger and fulfillment of the SDGs.

## 5 Goal #7 – “Affordable and Clean Energy”

Goal #7 concerns the need to ensure access to affordable, reliable, sustainable, and modern energy for all. The United Nations estimated that nearly 733 million persons do not have access to electricity worldwide; based on the current trend, more than 670 million persons will still not have access to electricity in 2030. Although impressive progress in electrification was seen in the last three decades, this progress has slowed down during this decade [72].

Between 2010 and 2019, the annual energy-intensity improvement rate was 1.9%, but a much higher rate (3.2%) is required to achieve the SDGs target. This improvement rate will be only achieved if the world progresses in energy efficiency. The low energy efficiency observed in several economic sectors, such as agriculture, industry, and services, impedes access to energy for all. Due to energy restrictions and high prices, 2.4 billion people still used inefficient and polluting cooking systems in 2020 [72].

One of the main advantages of ultrasound application in fruit drying is reducing total processing time, especially the air-drying time. Air drying is energy-intensive and the main cost of dried fruit products; thus, shorter process time means lower energy requirements and costs.

**Fig. 9:**
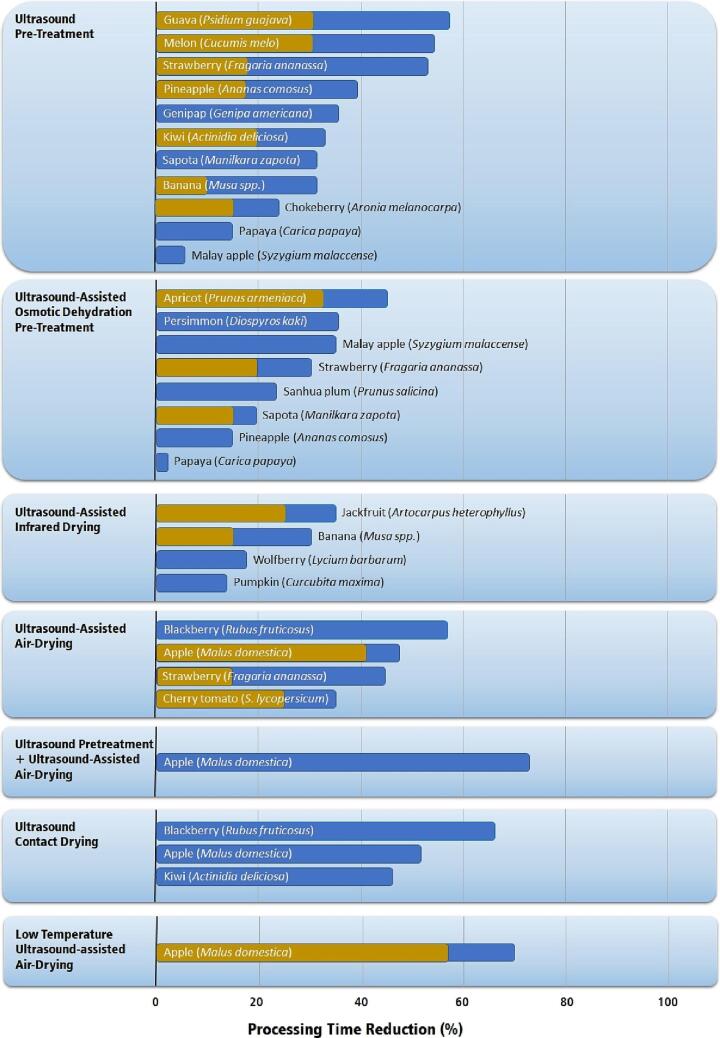
Percentual reduction in processing time for several fruits and ultrasonic drying processes compared to traditional air drying [24], [34], [38], [39], [81], [84], [86], [87], [88], [89], [90], [91], [92], [93], [94], [95], [97], [98], [99], [100], [101], [102], [103], [104], [105], [106]. Single-colored bars indicate unique values in the literature. Double-colored bars indicate the lowest (brown) and highest (blue) values reported in the literature.

The increase in water diffusion attained with ultrasound applications directly contributes to an increase in the drying rates and the reduction of processing time and cost. Fig. 9 shows the percentual reduction in the air-drying time achieved when applying ultrasound technologies.

Among all ultrasound techniques, ultrasonic pretreatment, ultrasound-assisted air drying, their combination, and ultrasound-assisted contact drying presented the highest percentual reduction in air-drying time, with the processing time of several fruits being reduced by more than 40%. Due to the energy-intensive characteristics of drying processes, even small reductions (greater than10%) are welcome and contribute to energy savings. Most case studies on ultrasound's application in drying processes successfully reduced energy consumption. This is an advantage of ultrasonic processes, which fulfills target 7.3, “*By 2030, double the global rate of improvement in energy efficiency*” of the SDGs.

The ultrasonic pretreatment considerably decreases the processing time and energy consumption for most fruits tested. The results were highly significant (greater than40% reduction) for the drying of guava (*Psidium guajava*) [87], melon (*Cucumis melo*) [97], and strawberries (*Fragaria ananassa*) [89], [98]. The ultrasound-assisted osmotic dehydration reduced the processing time, but its overall performance was lower than that of the ultrasonic pretreatment. The lower performance can be attributed to the fruit surface saturation with the osmotic solute, which reduces the apparent water diffusivity during the air-drying stage. The UAOD process was highly significant (greater than40% reduction) only for the drying of apricots (*Prunus armeniaca*) [101]. However, some fruits, such as the Malay apple (*Syzygium malaccense*) [91] presented a higher percentual time reduction applying UAOD.

Ultrasound-assisted air drying (UAAD) presents a performance equivalent to ultrasonic pretreatment. The UAAD process was highly significant (greater than40% reduction) for the drying of blackberries (*Rubus fruticosus*) [39], apples (*Malus domestica*) [104], and strawberries (*Fragaria ananassa*) [34]. The UAAD is an interesting technology because it applies ultrasound during air-drying, not requiring a pretreatment. However, scaling up this technology is more complicated than scaling up ultrasonic pretreatment. To date, the application of UAAD has been restricted to a lab scale. Ultrasonic waves tend to be disrupted by the air flow rate, and the vibrating chamber must be close to the fruit being dried. Further studies are required to scale up the UAAD equipment. Still, resources from the industry may be necessary to develop, build, and test a pilot-scale drier due to the high initial cost of this kind of development. Such need for further development and high cost for implementation probably unable this technology to achieve SDG #2 “End Hunger” by the timeframe established by the United Nations. However, it may contribute to SDG #2 in the long term.

Ultrasound-assisted contact air drier showed excellent performance [38], [39], [105] reducing the drying time and, therefore, the process cost. The higher performance of this technology is due to the direct contact and transfer of ultrasonic energy from the transducer to the fruit. All other technologies rely on an indirect transfer of ultrasonic energy from the transducer to the fruit. This technology can be more easily scaled than the UAAD since it depends mainly on increasing the surface area and the number of transducers.

Ultrasound applications consume little energy compared to the energy required to heat air. In most cases, the energy consumption of ultrasound is lower than 2% of the total energy consumption for drying. When pretreatments are used, the pretreatment time should be optimized to have the lowest air-drying time. Optimal pretreatments are usually carried out for 20 to 45 min [81], [89], [107] and depend on the fruit being processed. Optimization studies are extremely important for the UAOD pretreatment because overexposure to the osmotic solution may saturate the fruit surface with solute, blocking the fruit pores and microscopic channels formed by ultrasound, resulting in a reduction of the apparent water diffusivity.

## 6 Goal#3 – “Good health and Well-Being”

Goal #3 concerns the need to ensure healthy lives and promote well-being for all at all ages. Food is directly linked to health, being a good source of vitamins, bioactive compounds, sugars, fibers, and other essential nutrients.

Most fruits have important nutritional properties with high therapeutic potential. Some fruits, such as blueberry (*Vaccinium myrtillus*), blackberry (*Rubus fruticosus*), and cranberry (*Vaccinium oxycoccos*), are even considered functional foods due to their content of bioactive compounds. Along with sugars, fats, amino acids, and fibers, fruits have phenolics, vitamins, anthocyanins, ellagitannins, flavonoids, minerals, carotenoids, betalains, and other nutritional compounds. Consumption of fruit has been correlated with several health benefits, such as anti-cancer, anti-diabetic, anti-atherogenic, anti-thrombotic, anti-apoptotic, cardio-protective, neuro-protective, and anti-inflammatory roles [108], [109], [110], [111].

Drying does not significantly change the sugar content in fruits. Removal of water concentrates the fruit sugars, but the mass of sugar per total dried mass does not change substantially. However, pretreatments do change sugar content. Ultrasonic pretreatment carried out in potable or distilled water removes sugars from fruits due to the osmotic pressure difference and sponge effect. On the other hand, ultrasound-assisted osmotic dehydration increases sugar content in fruits due to the osmotic gradient between the fruit and the osmotic solution.

Several fruits have an amount of sugar equivalent to a cup of sugar-sweetened soft drinks. The difference is that fruits have several other beneficial compounds that soft drinks do not have (empty calories). All this sugar ends up in the dried fruit, which will have a considerable mass of sugar. High sugar intake has been associated with several cardiovascular diseases, insulin resistance, kidney disease, obesity, hyperglycemia, inflammatory bowel disease, and other health problems [112], [113]. Thus, the reduction of sugars in high-sugar-content foods can be interesting from a health point of view.

**Fig. 10:**
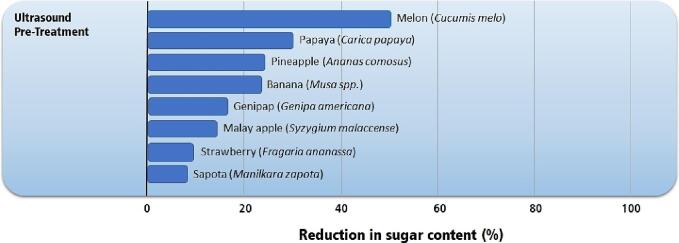
Reduction in sugar content in fruits by applying ultrasound pretreatment [81], [83], [84], [89], [90], [91], [107].

The ultrasonic pretreatment showed to be a promising technology for reducing sugar content in fruits. Fig. 10 presents the reduction in sugar content attained by applying ultrasound pretreatment. Significant sugar reductions (greater than30%) have been reported for melons, papaya, pineapple, and banana [81], [84], [86]. The sugar content decrease depending on the fruit tissue structure and pretreatment time. A maximum sugar loss is usually observed, and prolonging the processing time does not guarantee higher sugar losses, which tend to occur in the first 20 to 40 min of pretreatment.

As the ultrasound pre-treatment tends to remove sugars from the fruit, some consumers with a “sweet tooth” reject this kind of product because of its lower sweetness. To meet the expectations of these consumers, a dual-stage sugar substitution can be carried out. In this process, first, sugar is removed from the fruit using ultrasound pre-treatment. Then in the second stage, the fruit is immersed in a solution containing a low-calorie sugar (stevioside or rebaudioside) or other sweetener and subjected to ultrasound to incorporate it into the fruit [114], [115]. The second stage is usually carried out for 5 to 10 min since the sweetener has much higher sweetening power than the fruits’ natural sugars.

**Fig. 11:**
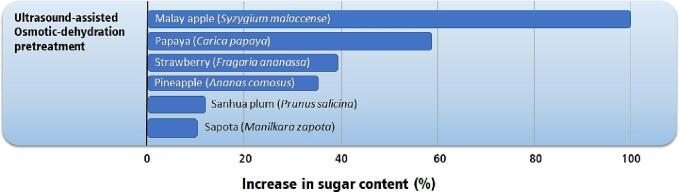
Highest sugar gain in fruits by applying ultrasound-assisted osmotic dehydration pretreatment [83], [84], [86], [91], [95], [99], [116].

The ultrasound-assisted osmotic dehydration may increase the effective water diffusivity, but its main disadvantage is increasing the sugar content in the dried fruit. Fig. 11 presents the sugar content increase observed after the UAOD pretreatment application. Increases higher than 30% in sugars are commonly achieved, leading to a sugar-rich product [83], [84], [86], [95], [99], [116]. For some fruits, such as papaya (*Carica papaya*) and Malay apples (*Syzygium malaccense*), the increase can be between 50 and 100% [91], which can be considered excessive. Sugar gain increases with increasing osmotic solution concentration and processing times. From a health perspective, UAOD should be avoided despite the significant cost reduction compared to conventional air drying.

Many studies have been carried out since the early 2000 s regarding the bioactive content in fruits and fruit products and their antioxidant capacity. Although many studies report the phenolic content, antioxidant capacity, and other bioactive compound content of dried fruits involving ultrasound technologies, some do not present the values for conventional drying. Thus, only sometimes is it possible to compare the traditional and ultrasonic processes and determine the contribution of ultrasound.

**Fig. 12:**
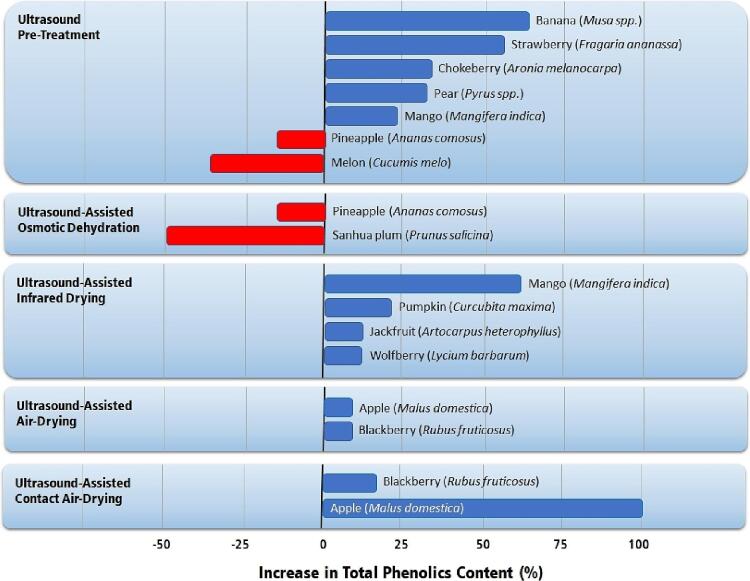
Highest increase in total phenolics content in fruits by applying ultrasound technologies compared to conventional air-drying [24], [38], [39], [92], [95], [97], [98], [99], [100], [102], [103], [104], [117], [118], [119].

Ultrasound technologies tend to produce dried fruits with higher phenolic content than dried fruit produced by conventional drying (Fig. 12); however, some opposite results have been reported. Studies with ultrasound pretreatment showed mixed results, with several fruits presenting higher phenolic content and some fruits presenting lower phenolic content [24], [97], [98], [99], [100], [117], [118], [119]. Ultrasound-assisted infrared drying, air-drying, and contact air-drying produce dried fruits with higher phenolic content than conventional air drying [38], [39], [92], [102], [103], [104], [118]. On the other hand, ultrasound-assisted osmotic dehydration tends to reduce the phenolic content of the fruit [95], [99].

The higher phenolic content of fruit subjected to ultrasound technology can have several causes. Most researchers attribute the higher phenolic content to the shorter exposure of the phenolics to then high or moderate temperatures (40 to 70 °C) applied during drying. Several phenolic compounds are thermolabile and therefore decay under heat, and shorter drying times result in lower phenolic decay than conventional air drying.

A second explanation regards a higher bioavailability of phenolics after ultrasound processing. Some phenolics are bonded to cell membranes, and some are located in the lipid layer of cell membranes. The ultrasonic energy transferred to the cells is enough to break the bond between the phenolics and cell membranes, generating more phenolics in their free form. Cell breakdown during ultrasound application also transfers phenolics from the lipid layer to the cytoplasmatic medium. Thus, increasing the bioavailability of phenolics in the dried fruit.

The total phenolic content can increase due to the breakdown of tannins, which may form large amounts of monomeric phenolics [120]. Although this route has not been associated with dried fruit production, it’s a real possibility for tannin-rich fruits subjected to ultrasound pretreatment. Ultrasound pretreatment can activate the phenylpropanoid pathway due to increased hydrogen peroxide and hydroxyl radicals [121]. This pathway is activated in stress conditions and is triggered by increasing concentrations of hydrogen peroxide and hydroxyl radicals leading to the consumption of sugars and the production of phenolics in fruits. Usual ultrasonic processing times (20 to 45 min for bath ultrasound and 5 to 10 min for probe ultrasound) are enough to induce the production of phenolics in fruits, which can result in higher phenolic content [27], [90], [107], [122]. The activation of the phenylpropanoid pathway is not expected in ultrasound-assisted infrared drying, air drying, and contact air drying due to the much lower water content in the fruit surrounding, which significantly reduces the production of hydrogen peroxide and hydroxyl radicals.

The lower phenolic content observed in osmotically treated fruits is attributed to the lixiviation of phenolics. During the osmotic treatment, phenolics may be carried by the water leaving the fruit towards the osmotic solution reducing the phenolics content in the fruit.

There are still very few studies on the effects of ultrasound on individual phenolics, and most studies that reported the phenolics profile in fruits still need to deeply discuss the impact of ultrasound on phenolics. Overall, small phenolic molecules, such as ellagic acid, gallic acid, protocatechuic acid, synaptic acid, caffeic acid, and others, are resistant to ultrasound applications and do not significantly decay when subjected to ultrasound [18]. Larger phenolic molecules, such as anthocyanins and flavonoids, are more susceptible to ultrasound and present a higher degree of decay. The decay of phenolics observed in dried fruits can be attributed mainly to the thermal effect rather than the ultrasonic effect.

**Fig. 13:**
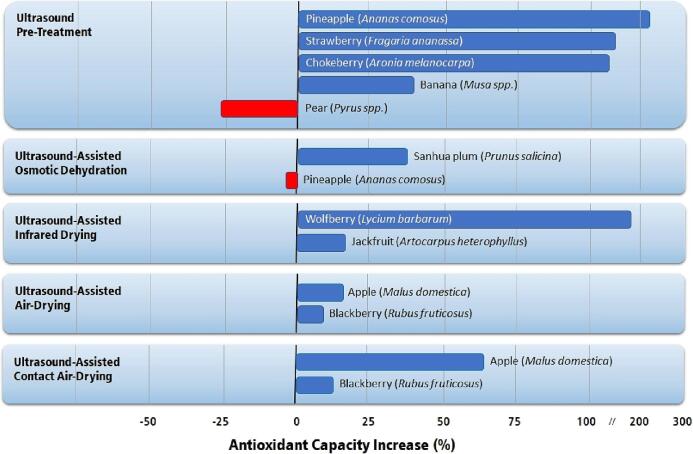
Highest increase in antioxidant capacity in fruits by applying ultrasound technologies compared to conventional air-drying [24], [38], [39], [92], [95], [98], [99], [100], [102], [104], [119].

Following the same trend observed with phenolics, the antioxidant capacity of many ultrasound-treated fruits usually increases (Fig. 13). The correlation between phenolic content and antioxidant capacity is well known. Thus, the increase in antioxidant capacity is expected due to the rise in phenolic content.

According to the data available in the literature, very high increases in antioxidant capacity are observed after ultrasound pre-treatment [24], [98], [99], [100] and ultrasound-assisted infrared drying [102]. An interesting aspect of ultrasonically treated fruits is that increases above 100% in the antioxidant capacity are not rare, and the treated product can be considered a functional food due to its improved properties.

Several reports indicate a significant decrease in the antioxidant capacity and phenolic content of fruits subjected to ultrasound. These reports usually refer to ultrasound pretreatments or ultrasound-assisted osmotic dehydrations that apply improper conditions in the use of ultrasound. Ultrasound produces a reasonable concentration of reactive oxygen species that oxidize many bioactive compounds leading to a decrease in antioxidant capacity and phenolic content. Although long treatment times should be avoided, many studies apply sonication for over 45 min. Under these conditions, the concentration of ROS will probably degrade a great part of the fruit’s bioactive compounds without improving the mass transfer rates or water diffusivity during the drying period. Most successful applications of ultrasound are carried out under 30 min in bath ultrasound and 10 min in probe ultrasound. Longer processing times may slightly improve mass transfer rates but will degrade considerably the nutritional and sensory aspects of the product.

**Fig. 14:**
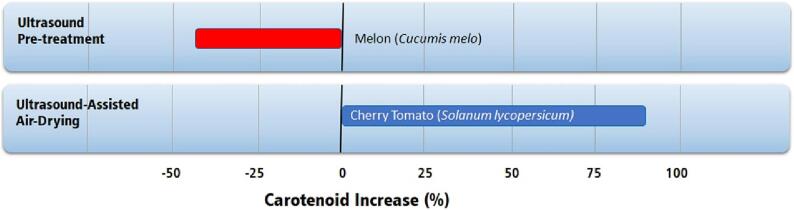
Highest increase in total carotenoids in fruits by applying ultrasound technologies compared to conventional air-drying [94], [124].

Although ultrasound technology has been studied in drying processes for at least 20 years, there are several gaps in knowledge regarding the effects of ultrasound on several classes of bioactive and nutritional compounds, such as carotenoids, anthocyanins, flavonoids, vitamins, aroma compounds, and others. Some articles [94], [123], [124] have addressed these classes of compounds, but it is impossible to pinpoint a general trend. For example, studies on carotenoid content after ultrasound drying showed contradictory results (Fig. 14).

## 7 Goal #8 – “Decent work and economic Growth”

The SDG #8 is related to decent work and economic growth. Within Goal #8, ultrasound drying of fruits can be connected to two targets: Target 8.2 “*achieve higher levels of economic productivity through diversification, technological upgrading, and innovation, including through a focus on high-value added and labor-intensive sectors*”, and Target 8.3 “*promote development-oriented policies that support productive activities, decent job creation, entrepreneurship, creativity, and innovation, and encourage the formalization and growth of micro-, small- and medium-sized enterprises, including through access to financial services”.*

**Fig. 15:**
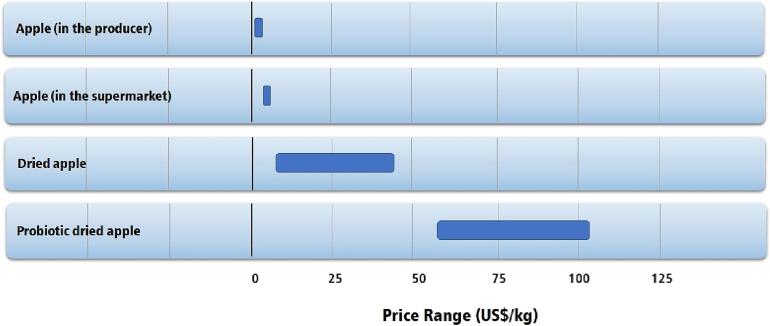
Average prices of apples and processed apple products (US$/kg in December/2022) [134], [135], [136].

Fruit drying is labor-intensive. Small, medium, and large fruit drying facilities rely on people to select and cut fruit, operate machinery, transport fruit and products, perform quality control, packaging, and many other functions within the facility. Bigger facilities usually have a higher degree of automatization but still employ many people. Small facilities typically rely on manual labor and are very labor-intensive. Although dried fruits are not considered a high-value-added product, new products developed with the use of ultrasonics are considered high-value-added products, such as probiotic dried fruits [125], [126], [127], prebiotic dried fruits [128], [129], [130], enriched dried fruits [131], [132], [133], low-calorie dried fruits [114], [115]. Fig. 15 presents a comparison of the price of several dried fruit-based products.

As new processes and technology are directed toward a product, it usually gains value and market price. Although it may look that dried fruits are much more expensive than their unprocessed counterparts, they have a higher density of solids and nutrients per mass. Dried fruits have a much longer shelf life than natural fruits, which is an important feature when considering the distribution of dried fruits to places where undernourishment is a severe problem.

As industries incorporate more technology, they tend to reduce the number of workers, which may induce higher unemployment and lower living conditions. However, this is different from the food industry, which usually is kept as labor intensive. Incorporating new technologies increases productivity and allows the development of high-added-value products. Technological products generally require workers with higher qualifications and, therefore, higher salaries. Economic growth comes through the production of high-added-value products and higher income for the workers and their community. Decent workplaces come with technology since workers with higher qualifications usually demand better work conditions.

Ultrasound technology intensifies drying and improves the impregnation of some bioactive compounds and microorganisms. Thus, the technology can be applied to produce high-value functional dried fruits.

The production of dried probiotic food products is challenging because most microorganisms do not support heat for long periods. As such, most probiotic products are sold in liquid form, especially dairy products. Spray-drying of liquid food with probiotics enables the production of several powdered probiotic products. However, these products must be reconstituted in some liquid to be consumed. The availability of ready-to-eat solid probiotic products is still scarce.

Dried apples (*Malus domestica*) and jackfruit (*Artocarpus heterophyllus*) incorporated with *Lactobacillus casei* have been successfully produced using ultrasound technology [137], [138]. The significant reduction in drying time obtained by ultrasound pre-treatments and ultrasound-assisted air-drying allows a better chance for microorganism survival. The process was probably successful because it combined a heat-tolerant microorganism (*Lactobacillus casei*) and a fruit that dries reasonably fast and that is significantly affected by ultrasound in reducing its drying time. Thus, this process may also be successful with other probiotic microorganisms, such as *Saccharomyces cerevisiae* var. *boulardii, Enterococcus faecium, Bacillus coagulans,* and other fruits, such as guava, strawberry, blackberry, melon, pineapple, and other fast-drying fruits.

Ultrasound pre-treatment has also been used to produce fortified or enriched dried products, such as iron-fortified pineapple chips [133], calcium-fortified melon snacks [132], and phenolic-enriched mango snacks [131]. This simple procedure relies on the sponge effect caused by ultrasonic waves to improve the incorporation of minerals or bioactive compounds. The fruit is immersed in an osmotic solution containing a soluble form of minerals, bioactive compounds, or microencapsulated compounds and subjected to ultrasound for 10 to 60 min. The mineral or bioactive compound is then incorporated into the fruit due to the concentration gradient between the compound in the solution and the fruit. Ultrasound application generates microscopic channels in the fruit, facilitating the entrance of the target compound. The sponge effect also improves the incorporation of the compound into the fruit. Another advantage of the ultrasonic method is that the target compound may be incorporated deep into the fruit and not only on its surface.

In the case of hydrophilic materials, such as encapsulated ferrous sulfate for iron fortification, the osmotic solution is based on ethanol rather than water [133]. Although the fruit will contain a reasonable amount of ethanol after osmosis, ethanol will be almost entirely removed during the drying stage since it has a lower vapor pressure than water. Ethanol residue accounts for<0.5% on a dry basis [133]. Despite the low content of ethanol, the dried fruit still contains alcohol which can be a problem for people addicted to alcohol and due to religious aspects. An example is Halal Foods. Generally, an ethanol content of up to 0.5% is considered acceptable in Halal food ingredients, but this acceptable limit differs among countries [139], [140], [141]. While the Thai-FDA admits 0.5% of added-in or natural ethanol in food, the Islamic Food and Nutrition Council of America accepts only 0.1% of ethanol [142]. A global standard for the allowable limit of ethanol in Halal-certified food does not exist, and in some countries, the *Al Istihlak* concept is applied and considers that food will not lose its Halal status if ethanol is not detectable by taste, smell, or sight [139]. However, in some countries, there are limits to naturally formed ethanol, but all added industrial ethanol is prohibited [141]. Therefore, although ethanol enhances mass transfer and reduces drying costs, its application should be labeled, and the product cannot be consumed by everyone.

The ultrasonic processes showed to be between 10 and 20% more efficient than the conventional method for iron incorporation and increased the iron content in pineapples by slightly more than 1000%, which is a significant value [133]. The same trend was observed for calcium-fortified melons and phenolic-enriched mangoes. The efficacy of the ultrasonic process was 7% higher than the conventional method for producing calcium-fortified melons. The resulting product contained 170% more calcium and 40% more phenolics than the non-fortified version. In both latter case studies, vacuum incorporation performed better than the ultrasonic process [131], [132].

## 8 Goal #9 – “Industry, Innovation, and Infrastructure”

Goal #9 regards building resilient infrastructure, promoting inclusive and sustainable industrialization, and fostering innovation. According to the United Nations, higher-technology industries are far more resilient in crises than their lower-tech counterparts.

A few large and thousands of medium-sized and small local industries dominate the dried fruit market. Large industries employ conventional and high technology but tend to produce few products, mainly raisins (dried grapes), dried dates, apricots, cranberries, and nuts. The medium and small-sized industries usually have a more extensive portfolio of regional fruits but rarely employ high technology. Within SDG #9, these medium and small-sized industries must embrace new technologies to provide good products at lower prices through higher productivity.

Ultrasonic baths, ultrasonic probes, and air-borne ultrasound systems dominate ultrasound technologies for fruit drying. Ultrasonic baths are cheap, easy to scale up, and can be found in many volumes, from liters to thousands of liters. The disadvantage of bath ultrasounds is their low power density, but most ultrasonic pre-treatments are carried out at low power densities (<100 W/L). Due to the low power densities, most bath ultrasound applications require long processing times. Ultrasonic probes are much more expensive than ultrasonic baths but provide a much higher power density (greater than1000 W/L); however, high power densities tend to degrade too much of the fruit tissue resulting in low-quality dried fruit. The ultrasonic probe can be scaled up, but there is a limit to the current technology. Lab-scale ultrasonic probes output 500 to 700 W, while industrial-scale ultrasonic probes go as far as 4000 W and can only be used for short periods without overheating. Air-borne ultrasound systems are still rare for fruit drying, and most systems are custom-built for research. The most significant disadvantage of air-borne ultrasound is its scale-up limitations since the ultrasonic waves tend to disrupt when distant from the samples being treated (greater than20 cm) and when high air velocities are employed (greater than4 m/s).

In the face of the advantages and disadvantages of each main ultrasound technology, most applications rely on ultrasound pre-treatments in ultrasonic baths followed by traditional drying techniques, such as oven [20], [107], forced convective [27], [28], [37], [143], [144], fluidized bed [145], [146], microwave [147], [148], [149], infrared [92], [93], [102], [118], vacuum-assisted [150], [151], and freeze-drying [152], [153], [154].

In the last ten years, no disruptive innovation in the ultrasound-assisted drying of fruits has been published. Most research consisted of incremental advances and case studies in ultrasound-assisted drying. Research has mainly focused on using ultrasonic pre-treatment (UPT) or ultrasound-assisted osmotic dehydration (UAOD) followed by traditional drying or a combination of techniques.

Innovation in the ultrasonic pre-treatment also was the focus of a few research. The multi-mode dual-frequency ultrasound pretreatment has been proposed by Xu et al. [155]. This technique involves applying two ultrasonic frequencies during the pretreatment, either at the same time or sequentially switching pulses of the two frequencies. According to their results, the dual-frequency overcomes some problems of the single-frequency application, such as directional sensitivity and uneven energy propagation. Furthermore, the sequential mode switching pulses of two frequencies resulted in shorter drying times and better quality of the dried fruit than the simultaneous application of two frequencies.

Several researchers have studied improvement of the drying process but mainly focused on post-ultrasound pretreatment rather than on innovation in ultrasonic technology. For example, Jiang et al. [26] studied the application of UAOD in strawberries before freezing them and subjecting the frozen strawberries (*Fragaria ananassa*) to pulsed fluidized bed microwave drying. Their process resulted in a processing time 45% lower than normal freeze-drying. Although the improvement was significant, their process is over-complicated to be applied to medium and small-sized industries. However, it may be a suitable process for large-sized industries with enough investment grants.

Ultrasound pretreatment in ultrasonic baths followed by traditional drying techniques has reached a technological maturity stage allowing its industrial application, especially in fruit drying. Still, we do not see industries using ultrasound on a large scale, except during the washing stage. Several reasons are behind this reality, including lack of knowledge of new processes and technologies, cost to implement ultrasonic processes in medium and small-size industries, lack of consultants to start up and tune the process, lack of interest in using more advanced technologies, and fear of changing a process that is work.

In conclusion, although ultrasound-assisted drying has been proven to be successful and economically viable, its spread into medium and small-size industries has been very slow. Unless governments give proper incentives for medium and small-size industries to develop and incorporate new technologies, SDG #9 will not be fulfilled regarding fruit drying.

## 9 Goal #12 – “Responsible consumption and Production”

Goal #12 concerns the need to ensure sustainable consumption and production patterns. According to the 2030 Agenda, unsustainable consumption and production patterns are the root cause of climate change, biodiversity loss, and pollution.

Too much food is being lost or wasted in every country every day. According to the United Nations, 13% of the world’s food is lost after harvesting and before reaching retail markets. These losses occur during harvest, transport, storage, and processing. Another 17% of food is wasted at the consumer level, including our houses, grocery stores, and restaurants. Thus, 30% of all food produced is never eaten [72].

Most consumers do not like to buy or eat fruits that present little defects or do not present an ideal color or shape. In developed countries, supermarkets select fruits and only sell near-perfect fruits, discarding fruits with few defects. As such, part of the product is not even sold. Consumers in under-developed countries accept fruits with few defects or less perfect color and shape, but too many fruits are still discarded.

Good fruits less prone to be sold to consumers can be processed and dried, increasing their shelf-live and overall appearance. Ultrasound-assisted drying may help improve some important sensory aspects of dried fruits, such as color, which has to remain stable for long periods.

The color of dried fruits is affected by enzymatic and non-enzymatic browning. Studies on enzyme inactivation by ultrasound have been carried out for more than 40 years, and many enzymes can be totally or partially inactivated [156], [157]. Studies on the reduction of non-enzymatic browning by ultrasound application are more recent and have concluded that ultrasound can successfully reduce non-enzymatic browning, improving color stability [99], [119], [158], [159].

Enzymatic browning of fruit products is mainly caused by two enzymes: polyphenol oxidase (PPO) and peroxidase (POD). Other enzymes, such as ascorbate peroxide (PAX) and lipoxygenase (LOX), can also contribute to changes in color in citrus and fatty acid-rich products, for example.

**Fig. 16:**
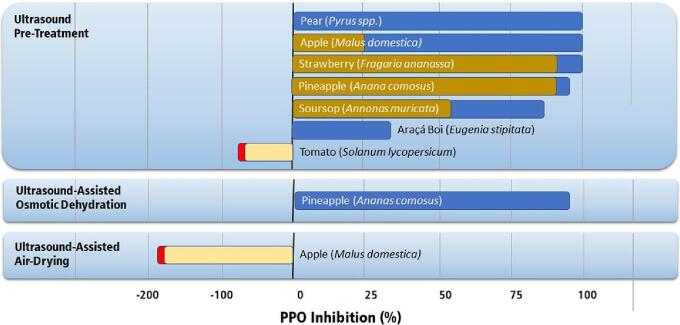
Highest inhibition of PPO in fruits by applying ultrasound technologies [25], [99], [156], [157], [160], [161], [162], [163], [164]. Single-colored bars indicate unique values in the literature. Double-colored bars indicate the lowest (brown and yellow) and highest (blue and red) values reported in the literature.

**Fig. 17:**
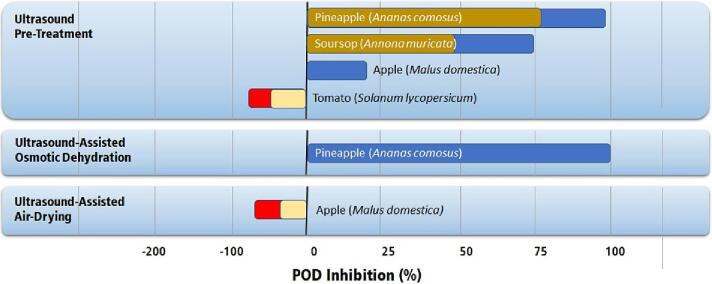
Highest inhibition of POD in fruits by applying ultrasound technologies [25], [99], [156], [157], [160], [161], [162], [163], [164]. Single-colored bars indicate unique values in the literature. Double-colored bars indicate the lowest (brown and yellow) and highest (blue and red) values reported in the literature.

Inactivation of PPO and POD is usually carried out by immersing the fruit in an ultrasonic bath as in the ultrasonic pre-treatment. Most studies report a partial inactivation of PPO and POD [25], [99], [156], [157], [160], [161], [162]. Thermosonication increases the inactivation process's efficacy, reducing these enzymes' residual activity. On the other hand, some studies report an enzymatic activity increase after ultrasound processing [163], [164]. Fig. 16, Fig. 17 present the percentual inhibition of PPO and POD reported in the literature for fruits subjected to the ultrasonic treatment.

Several mechanisms have been proposed to explain the inactivation of enzymes by ultrasound technology. Micro-jets or micro-streaming generated by ultrasound application in a bath or probe ultrasounds can interrupt polypeptide Van der Waals interactions and hydrogen bonds, leading to enzyme inactivation [165]. The free radicals produced by ultrasound react with the enzyme’s disulfide bonds, destabilizing its conformation. The free radicals can also oxidize the amino acids tryptophan, histidine, cysteine, and tyrosine, resulting in a loss of enzyme activity [165]. The increase in process temperature creates a synergic effect of ultrasound and heat that lead to enzymatic dissociation of the prosthetic group, resulting in conformational changes in the secondary and tertiary structures of both PPO and POD [166], [167].

Ultrasound does not always inactivate these enzymes. Sometimes, PPO and POD are activated by ultrasound processing due to the increased concentration of reactive oxygen species, which activates both PPO and POD. Inactivation or activation of oxidases depends on the fruit tissue structure and the treatment conditions. When not enough energy is provided by ultrasound, the enzymes may not suffer conformational changes and therefore do not inactivate. Also, the enzymes will activate if the concentration of reactive oxygen species formed during sonication is below what the enzyme can process. They will inactivate mainly if an excess of reactive oxygen species is produced, giving a higher probability for these species to react with the enzyme bonds changing their chemical and physical structure.

Changes in color during fruit drying are relatively common due to enzymatic and non-enzymatic browning. The effect of ultrasound on enzymatic browning can be easily accessed by measuring the activity of the browning enzymes. The determination of the rate of non-enzymatic browning is more complex, depending on the chromatographic analysis of compounds produced during non-enzymatic browning, such as hydroxymethylfurfural (HMF). The literature lacks papers on non-enzymatic browning related to fruit drying, especially ultrasound fruit drying.

**Fig. 18:**
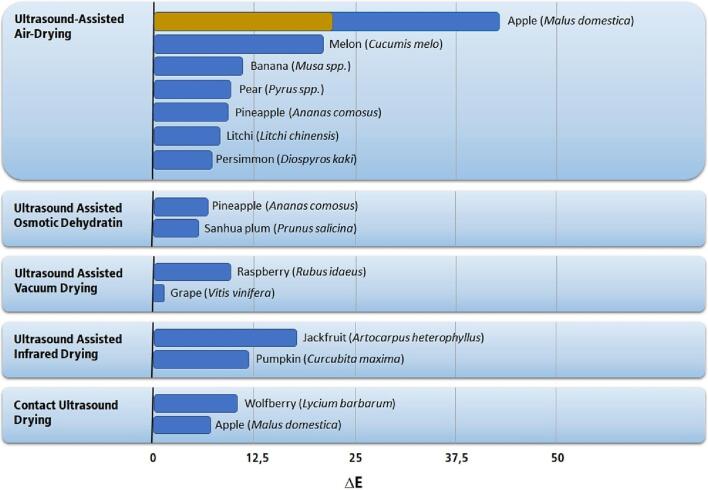
Highest changes of color in fruits by applying ultrasound technologies [24], [25], [38], [92], [95], [97], [99], [102], [103], [119], [147], [148], [158], [159], [168]. Single-colored bars indicate unique values in the literature. Double-colored bars indicate the lowest (brown) and highest (blue) values reported in the literature.

The information available for this process consists of the measurement of color change caused by drying, which combines both enzymatic and non-enzymatic browning. Fig. 18 presents the change of color for fruits subjected to the ultrasonic treatment.

In most fruits, the color changes mainly affect the luminosity (L), redness (a), and hue (h) parameters. An increase in luminosity is usually viewed as positive. Changes in the redness or hue are considered negative because they refer to excessive browning. Color change during the drying process is unavoidable, and it can be reduced by ultrasound pretreatment or other methods, but not entirely stopped. Most sonicated fruits present color changes (ΔE) lower than 10, which can be considered reasonable since the human eye can only perceive changes more significant than 3 or 5 for some color spectra (blue-green) and older people [169], [170], [171], [172]. Studies in dentistry have shown that changes as little as DE 1 can be perceived by 50% of consumers, but this value only refers to white colors, which is rarely the case with fruits [173].

The color change resulting from ultrasound-assisted drying processes is usually lower than the ΔE 10 barrier, but fruits such as apples (*Malus domestica*), jackfruit (*Artocarpus heterophyllus*), and melons (*Cucumis melo*) present higher color changes. In common, they do not have colored pigments; thus, browning is more perceivable. Furthermore, apples have a high content of highly active browning enzymes, which makes it challenging to maintain their original color due to only partial inactivation of these enzymes. Among the ultrasonic processes, direct contact ultrasound drying is the only one that reduced significantly apple browning, probably due to higher inactivation of the browning enzyme since the fruit is constantly subjected to ultrasound during the entire drying time.

Thus, ultrasonic pretreatment can improve product safety, reduce drying time, increase shelf-life, and usually does not compromise significantly sensory aspects like color. This combination leads to responsible production and responsible consumption since consumers can keep ultrasound-assisted dried fruits for long periods, considerably reducing food waste.

Besides color, the aroma and taste of the dried product must meet consumers’ expectations. The effects of ultrasound on aroma and taste are not consolidated since very few studies have been done on this theme. The reduction in drying time attained by ultrasound processing has a positive effect in retaining a higher concentration of volatile compounds in the dried product [99]. Therefore, the ultrasonic-treated fruit has a more intense aroma than the fruit dried without ultrasound. However, it is less severe than the fresh product because part of the volatile compounds is lost during the ultrasonic pretreatment and drying. Ultrasound-assisted osmotic dehydration tends to retain more volatile compounds than ultrasound pretreatment in water. The ultrasound pretreatment in ethanol may result in a higher intensity of the notes related to alcohol because the evaporation of ethanol incorporated by the fruit is incomplete, as in pineapples [99].

The effect of ultrasound on the taste depends on the type of ultrasound technology implemented. Ultrasonic pretreatment in water tends to reduce sugar content resulting in less sweet dried fruit than natural fruit. Ultrasound-assisted osmotic dehydration in sugar solutions tends to increase sugar content leading to sweeter products. A study with strawberries subjected to US pretreatment reported that the dried US-pretreated strawberries presented higher bitterness, sweetness, umami, and saltiness than the traditionally dried strawberries and lower sourness [152]. The higher sweetness and lower sourness were correlated with the higher sugar and lower organic acid content of the US-pretreated strawberries compared to the traditionally dried strawberries. The US-pretreated fruit lost organic acid during the pretreatment, while the lower drying time resulted in lower caramelization of sugar and, therefore, higher sweetness. The increase in bitterness and umami may be related to chemical reactions between the fruit amino acids and reactive oxygen species produced during ultrasound application. Taste and aroma attributes were investigated for lulo fruit (*Solanum quitoense* Lam.) subjected to contact-ultrasound air-drying [174]. Contact ultrasound did not alter the cooked fruit aroma resulting from hot air drying. The taste of lulo fruit was considered less sweet and too acidic, caused by the caramelization of sugars with a consequent increase in the acid-to-sugar ratio. However, there is a huge lack in the literature on a high-quality discussion of changes in aroma and taste caused by ultrasound-assisted drying technologies that correlate the chemical modifications to its outcomes regarding aroma and taste descriptors.

Current knowledge does not allow a good evaluation of how ultrasonic processes affect aroma and taste. Most reports are subjective, and not all were done with the recommended number of panelists. Thus, their scientific value is questionable. On this point, it is still uncertain how ultrasonic processes can help toward responsible consumption.

## 10 Goal #17 – “Partnerships for the Goals”

Goal #17 refers to “strengthen the means of implementation and revitalize the global partnership for sustainable development”. Targets 17.7 and 17.8 concern science and technology. Target 17.7 stimulates sustainable technologies' development, transfer, dissemination, and diffusion, while Target 17.8 asks for funding to promote science, technology, and innovation.

Ultrasound technology for fruit drying is no longer novel since ultrasonic processes were proposed in the late 1990 s and were well-developed during the 2000 s. In the past 15 years, incremental research has further developed ultrasound-assisted fruit-drying processes.

Despite being a consolidated technology, there are still many opportunities for studies related to fruit drying. Most studies in the early stages of technology development focused on the physical phenomenon and engineering aspects of ultrasound-assisted drying. The physical effects of cavitation, jet-streaming and sponge effect on mass transfer and fruit tissue have been well established. Several engineering solutions have been proposed, such as the application of ultrasound baths and probes, contact ultrasound, air-borne ultrasound, and multi-frequency ultrasound, and the association of these technologies with other conventional and non-conventional air-drying processes. Optimization of the processes and energy conservation has been the focus of several research.

However, the chemical and biochemical aspects of the ultrasonic process for fruit drying are still in great need. During the 2000 s and 2010 s, many studies addressed how ultrasound affected phenolics, anthocyanins, carotenoids, vitamin C, sugars, and some organic acids. Except for vitamin C, sugars, and some organic acids, most studies reported the results of lumped assays for phenolics, anthocyanins, and carotenoids. Although the knowledge of total phenolics, anthocyanins, and carotenoids content is valuable, it does not allow a complete understanding of the chemical mechanisms involved in the process, the natural protective mechanisms of fruits against the reactive oxygen species (ROS) formed during ultrasound application and the reaction selectivity for each kind of bioactive compound. Some information and possible mechanisms can be correlated with studies on ultrasound applications in fruit juices and other processes. However, the effects of ultrasound and ROS during drying still need to be fully understood. It is very common to read that the impact of ultrasound need to be evaluated for each fruit, not being possible to generalize or predict what will happen. Thus, there is still a significant gap in the knowledge regarding the chemical effects of ultrasound.

Studies on changes in aroma and taste are rare, despite aroma and taste being major sensory characteristics that drive the will of consumers to buy a given product. More studies correlating operating conditions with the chemical changes in aroma and taste compounds and their descriptors are required.

SDGs #2 and #3 concerning zero hunger and good health and well-being require that food brings health and fulfillment. Healthy dried fruit products should have many bioactive compounds, such as vitamins, carotenoids, pro-carotenoids, flavonoids, polyphenols, and others. More in-deep studies on the chemical changes induced by ultrasound applications must be conducted. Simple questions, such as how much vitamin E remains in dried avocados, how much lycopene remains in dried guavas, or even what happens to vitamin E in fruits subjected to ultrasound, cannot be answered with reasonable certainty. Without this kind of study, it is impossible to guarantee that all ultrasound-assisted dried fruits will fully address SDGs #2 and #3.

## 11 Conclusions

Ultrasound technologies for fruit drying contribute toward many SDGs. The longer shelf life of dried products contributes immensely toward “Zero Hunger” (Goal #2), which is one of the main contributions of ultrasound technologies for fruit drying. The possibility of taking long shelf life healthy foods and well-being (Goal #3) to underdevelopment regions and countries where the population suffers from malnourishment is increased with ultrasound-assisted processes. Better food with fewer fruit losses directly impacts responsible production and consumption (Goal #12).

Ultrasound can be applied to the initial steps of fruit processing through washing and sanitization, reducing the need for sanitizing chemicals. With less toxic wastewater, ultrasound processing contributes to life on land and below water (Goals #14 and #15). The significant reduction in the processing time of the energy-intensive drying process contributes to saving energy (Goal #7).

The innovation brought by ultrasound technologies is directly related to Goal #9. Advanced technology, higher productivity, and lower costs in the processing industry call for better-paid workers, more decent workplaces, and, consequently, more economic growth (Goal #8). To accompany industrial innovation, more research and researchers are needed. Thus, more effective interaction between industry, universities, governments, and research centers will flourish (Goal #17).

Ultrasound-assisted fruit drying can impact many sustainable development goals, reducing hunger, delivering healthier foods, contributing to a better environment, reducing industrial costs, saving energy, and opening opportunities for more and more decent workplaces, that in the end, ultrasound technologies for fruit drying contributes toward one of the most important goals: No Poverty (Goal #1).

## Funding

This work was funded by Conselho Nacional de Desenvolvimento Científico e Tecnológico (CNPq) through the INCT Frutos Tropicias grant.

## CRediT authorship contribution statement

**Fabiano A.N. Fernandes:** Conceptualization, Data curation, Writing – original draft, Writing – review & editing. **Sueli Rodrigues:** Writing – original draft, Writing – review & editing.

## Declaration of Competing Interest

The authors declare that they have no known competing financial interests or personal relationships that could have appeared to influence the work reported in this paper.

## Data Availability

Data will be made available on request.
